# Draft Genome Sequence of *Gluconobacter frateurii* NBRC 103465, a Glyceric Acid-Producing Strain

**DOI:** 10.1128/genomeA.00369-13

**Published:** 2013-07-25

**Authors:** Shun Sato, Maiko Umemura, Hideaki Koike, Hiroshi Habe

**Affiliations:** Research Institute for Innovation in Sustainable Chemistry, National Institute of Advanced Industrial Science and Technology (AIST), Tsukuba, Ibaraki, Japana; Bioproduction Research Institute, National Institute of Advanced Industrial Science and Technology (AIST), Toyohira-ku, Sapporo, Hokkaido, Japanb; Bioproduction Research Institute, National Institute of Advanced Industrial Science and Technology (AIST), Tsukuba, Ibaraki, Japanc

## Abstract

*Gluconobacter frateurii* strain NBRC 103465 can efficiently produce glyceric acid (GA) from raw glycerol feedstock derived from biodiesel fuel production processes. Here, we report the 3.4-Mb draft genome sequence of *G. frateurii* NBRC 103465. The draft genome sequence can be applied to examine the enzymes and electron transport system involved in GA production.

## GENOME ANNOUNCEMENT

Acetic acid bacteria efficiently oxidize ethanol and various sugars and sugar alcohols, including d-glucose, glycerol, and d-sorbitol, owing to their incomplete oxidation and efficient secretion, termed oxidative fermentation (1). Of the family *Acetobacteraceae*, *Gluconobacter oxydans* is an important species widely used in the industrial production of dihydroxyacetone (DHA) and l-sorbose. Complete genome sequences of *G. oxydans* strains have been published (2, 3). We previously reported an efficient oxidative fermentation method for producing glyceric acid (GA) from glycerol using *Gluconobacter frateurii* strain NBRC 103465. The maximal 136 g/liter GA productivity (GA yield, 0.68 mol/mol-glycerol; d-GA enantiomeric excess, 72%) under optimized conditions was achieved using *G. frateurii* NBRC 103465 (4). GA was originally identified as a by-product of DHA production from glycerol by *G. oxydans* (5); however, GA is known to have some biological activities, such as d-GA-promoted acceleration of ethanol metabolism in rats (6). Despite these functions, GA production has been limited due to the high cost of catalytic synthesis.

The draft genome sequence of *G. frateurii* NBRC 103465 was generated at Agencourt Bioscience Co. (Beverly, MA) using a 454-GS-FLX-Titanium pyrosequencing technique. A paired-end 454 library with an average insert size of 3 kb was created and generated 1,163,877 reads and 317 Mb of read length, which provided an average 93.3× coverage of the genome. The 454 data were resolved to 7 scaffolds consisting of 3,375,892 bp. The sequence assemblies were constructed using optical maps. The optical maps and 7 scaffolds were compared to determine the relative order and orientation of each scaffold. Physical gaps and misassemblies were closed and corrected using custom primer walks, PCR amplification, and Sanger sequencing. Resultantly, the draft genome of *G. frateurii* NBRC 103465 consists of a circular chromosome (size, 3,205,714 bp; G+C content, 55.5%) and a plasmid (size, 190,377 bp; G+C content, 57.0%). Protein-coding genes were predicted using GeneLook 3 (7), identifying a total of 3,199 coding sequences, of which 3,010 are in the chromosome and 189 in the plasmid. A total of 57 tRNA genes and 14 rRNA genes were also identified.

The most significant feature of *G. frateurii* NBRC 103465 is its high GA productivity. In *G. oxydans*, we demonstrated that membrane-bound alcohol dehydrogenase (mADH) was involved in GA production from glycerol (4). Three genes encoding mADH subunits, namely, the large dehydrogenase subunit, the cytochrome *c* subunit, and a 15-kDa protein (8), were also identified in the *G. frateurii* NBRC 103465 genome. The similarities of these genes with homologs from *G. oxydans* 621H were 81.5%, 86.0%, and 82.7%, respectively. At least another 7 membrane-bound dehydrogenases involved in many oxidation reactions were annotated from the genome information. The draft genome sequence of *G. frateurii* NBRC 103465 may provide novel molecular information and increase our understanding of the efficient oxidation mechanism of glycerol to GA.

### Nucleotide sequence accession numbers.

The *G. frateurii* NBRC 103465 genome sequence was deposited as 11 contigs at DDBJ/EMBL/GenBank under accession no. BARL01000001 to BARL01000011. The version described in this study is the first version.
